# Progeny production in the periplasm of *Thermosipho globiformans*

**DOI:** 10.1007/s00792-017-0944-0

**Published:** 2017-06-02

**Authors:** Tomohiko Kuwabara, Kensuke Igarashi

**Affiliations:** 1 Faculty of Life and Environmental Sciences, University of Tsukuba, Tsukuba, 305-8572 Ibaraki Japan; 2 Bioproduction Research Institute, National Institute of Advanced Industrial Science and Technology (AIST), 2-17-2-1 Tsukisamu-Higashi, Toyohira-ku, Sapporo, 062-8517 Hokkaido Japan

**Keywords:** Eukaryogenesis, High-temperature microscopy, Peptidoglycan, Spheroid, Thermotogales

## Abstract

Thermotogales are rod-shaped, Gram-negative, anaerobic, (hyper) thermophiles distinguished by an outer sheath-like toga, which comprises an outer membrane (OM) and an amorphous layer (AL). *Thermosipho globiformans* bacteria can transform into spheroids with multiple cells concurrently with AL disintegration during early growth; the cell is defined as the cytoplasmic membrane (CM) plus the entity surrounded by the CM. Spheroids eventually produce rapidly moving periplasmic ‘progenies’ through an unknown mechanism. Here, we used high-temperature microscopy (HTM) to directly observe spheroid generation and growth. Rod OMs abruptly inflated to form ~2 μm-diameter balloons. Concurrently, multiple globular cells emerged in the balloons, suggesting their translocation and transformation from the rod state. During spheroid growth, the cells elongated and acquired a large dish shape by possible fusion. Spheroids with dish-shaped cells further enlarged to ~12 μm in diameter. HTM and epifluorescence-microscopy results collectively indicated that the nucleoids of dish-shaped cells transformed to form a ring shape, which then distorted to form a lip shape as the spheroid enlarged. HTM showed that ‘progenies’ were produced in the spheroid periplasm. Transmission electron microscopy results suggested that the ‘progenies’ represented immature progenies lacking togas, which were acquired subsequently.

**Electronic supplementary material:**

The online version of this article (doi:10.1007/s00792-017-0944-0) contains supplementary material, which is available to authorized users.

## Introduction

Thermotogales bacteria are rods that have a peculiar outer sheath-like envelope referred to as a toga (Huber and Stetter 1992), which consists of an outer membrane (OM) and an amorphous layer (AL) (Huber et al. 1986, 1989; L’Haridon et al. 2001; Kuwabara and Igarashi 2012). The toga forms a large periplasm at 1 or both ends of rods, which facilitates the identification of Thermotogales bacteria by optical microscopy (Huber and Stetter 1992). The OM is biochemically characterized in terms of some constituent proteins (Rachel et al. 1990; Engel et al. 1992; Engel et al. 1993), but the AL is not. To date, the AL is the only recognizable structure between the OM and cytoplasmic membrane (CM) that has been described by transmission electron microscopy (TEM) (Huber et al. 1986, 1989; L’Haridon et al. 2001; Kuwabara and Igarashi 2012). Peptidoglycans (PGs) in Thermotogales have not been identified in TEM images, although they have been characterized biochemically as lacking *meso*-diaminopimelic acid (Huber et al. 1986, 1990). Instead, the PGs contain l- and d-lysine residues at a ratio of 1:1 (Huber et al. 1986) at the third position of the peptide stem (Boniface et al. 2006; Boniface et al. 2009). It has not been clarified whether the AL contains the PG.

**Fig. 1:**
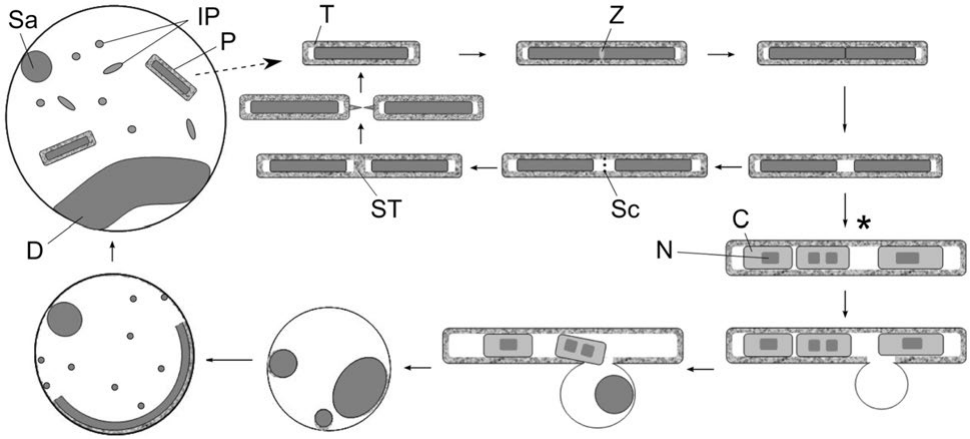
Reproductive cycle of *Thermosipho globiformans*, based on previous results (Kuwabara and Igarashi 2012) and data generated in this study. In rods, the FtsZ-type cell division may occur, as in other Thermotogales (Adams and Errington 2009), followed by the formation of a large periplasm between the divided cells (Kuwabara and Igarashi 2012). A scaffold is constructed in the periplasm, on which the septal toga is formed. Then, the daughter rods divide by pulling apart (Kuwabara et al. 2011). In the stationary phase, septal toga-forming activity declines and rods with multiple cells (*asterisk*) are formed (Kuwabara and Igarashi 2012). A subpopulation of rods triggers AL disintegration, but retains overall rod-like morphologies. Such rods transform into spheroids in fresh media. The spheroids grow and produce immature progenies, which maturate to rods in the periplasm. The immature progenies are depicted in the spheroid periplasm at different angles with respect to the plane. The broken line arrow indicates that rod ejection from the spheroid periplasm during growth is assumed, but has not been observed by HTM. The relative sizes of cell cycle components are not to scale. *C* cell, *D* dish-shaped cell, *IP* TEM-suggested immature progeny or optical microscopy-defined ‘progeny’, *N* nucleoid, *P* progeny rod, *Sa* satellite cell, *Sc* scaffold, *ST* septal toga, *T* toga, *Z* Z-ring

We recently developed anaerobic thermophile observation chambers (ATOCs) for high-temperature microscopy (HTM), which enabled the observation of thermophile growth at 65 °C (Kuwabara and Igarashi 2012). HTM, in combination with phase-contrast microscopy, epifluorescence microscopy, and TEM, revealed that septal toga formation occurs after cytokinesis is completed, differently from most Gram-negative bacteria whose cell and OM divide simultaneously. Because of this delay, the next round of cytokinesis may occur in daughter cells before septal toga formation occurs. Further delays of septal toga formation over multiple rounds of cytokinesis leads to the formation of multiple cells within the surrounding toga (Kuwabara and Igarashi 2012). “Cells in a chain,” observed previously with many Thermotogales species (Patel et al. 1985; Huber et al. 1986, 1989; Davey et al. 1993; Wery et al. 2001; DiPippo et al. 2009; Jayasinghearachchi and Lal 2011), were likely rods containing multiple cells, as represented in the illustration summarizing the morphological findings of this study (Fig. 1).

Many Thermotogales species transform into spheroids during the stationary phase (Patel et al. 1985; Huber et al. 1986, 1989, 1990; Jeanthon et al. 1995; Andrews and Patel 1996; Takahata et al. 2001; Balk et al. 2002). Spheroid formation is associated with disintegration of the AL (Kuwabara et al. 2011). Similar transformations have been reported with both Gram-negative and Gram-positive mesophiles (Lam et al. 2009; Leaver et al. 2009), in which mutations of genes involved in PG synthesis caused the transformation. Thus, the AL and PG have similar characteristics, both in terms of their intracellular localization and functions in maintaining rod-like morphologies. Among members of the Thermotogales order, *Thermosipho globiformans* is unusual in that this transformation also occurs during early growth phases (Kuwabara et al. 2011). Probably owing to this early transformation, its spheroids can produce ‘progenies’ within the periplasm (Fig. 1), which are defined here as rapidly moving miniscule vesicles containing nucleic acids (Kuwabara et al. 2011). However, exactly how rods transform into spheroids and how spheroids grow to become capable of reproduction remain unclear.

Here, we investigated the generation and growth of spheroids, as well as reproduction from spheroids, using a combination of HTM, phase-contrast microscopy, epifluorescence microscopy, and TEM. Our comprehensive observations of *T. globiformans* proliferation suggested that defective AL formation in rods led to transformation into spheroids and that AL formation occurred in ‘progenies’ to transform them into rods.

## Materials and methods

### Cultivation of *T. globiformans*

The *T. globiformans* MN14 strain was collected from an interface between anaerobic and aerobic environments, which was artificially generated at approximately 40 cm above a deep-sea hydrothermal vent, by deploying an in situ-cultivation device (Kuwabara et al. 2006). *T. globiformans* MN14 was isolated based on its natural ability to form spheroids each containing multiple cells during early growth phases (Kuwabara et al. 2011). It was deposited in the Japan Collection of Microorganisms (JCM 15059) and Deutsche Sammlung von Mikroorganismen und Zellkulturen repositories (DSM 19918). MN14 cells were maintained by anaerobic cultivation in 12 mL of Tc medium (pH 6.8) at 68 °C under a gas phase consisting of N_2_:H_2_:CO_2_ (80:10:10) up through the stationary phase, as described previously (Kuwabara et al., 2011). Tc medium contained (L^−1^) 25 g of NaCl, 0.33 g of KCl, 2.8 g of MgCl_2_·6H_2_O, 3.4 g of MgSO_4_·7H_2_O, 10 mg of NaBr, 0.3 g of K_2_HPO_4_, 0.25 g of NH_4_Cl, 0.025 g of Fe_2_SO_4_·7H_2_O, 10 g of elemental sulfur (S^0^), 10 mL each of trace minerals and vitamin solution (Balch et al. 1979), 3 g of yeast extract, 3 g of tryptone, 0.5 g of Na_2_S·9H_2_O, and 1 mg of resazurin. Tc medium devoid of Na_2_S was sterilized by Tyndallization, namely by autoclaving twice at 110 °C for 30 min within a 24-h interval, and then supplemented with Na_2_S for cultivation.

Cells were enumerated by simultaneous direct counting of rods and spheroids using the Live/Dead *Bac*Light Bacterial Viability Kit (hereafter referred to as “Live/Dead”; Life Technologies, Carlsbad, CA, USA), as described (Kuwabara et al. 2011). In cases where the cell density was too high to count cells, the samples were first diluted fourfold in 2% NaCl.

### ATOC preparation

Half-ATOCs were manually prepared by adhering a coverslip (30 mm × 30 mm, Matsunami Glass, Kishiwada, Japan), a piece of Pyrex tube (2 cm inside diameter × 1 cm height), and a septum prepared from a 0.5-mL sample tube (Kuwabara and Igarashi 2012). Heat-durable adhesive (Super X2; Cemedine, Tokyo, Japan) and toothpicks were used for adhesion in an anaerobic workstation, in which the gas phase was N_2_:H_2_:CO_2_ (80:10:10). A bottle containing Tc medium devoid of Na_2_S was heat-treated using an autoclaving device at 110 °C for 2 min and removed from the instrument when the temperature decreased to 96 °C. This manipulation was performed to decrease the gas content in the medium used for HTM. The bottle was swirled and placed in the workstation. When S^0^ was mostly settled, 2 mL of the supernatant was transferred to a sterilized test tube, supplemented with 10 μL of 10% Na_2_S·9H_2_O, and inoculated with 3–10 μL of a stationary phase culture; excess S^0^ and inoculum perturbed the HTM observations. Next, 1.5 mL of the inoculated medium was transferred into the half-ATOC, to which a glass slide (S1111, Matsunami) was adhered, producing a complete ATOC. The ATOC was removed after standing for more than 1 h in the workstation.

### Microscopy

The ATOC was inverted in a Petri dish and incubated in a programmable incubator, with the temperature increasing from room temperature to 33 °C for ≥10 h and then from 33 to 68 °C for 70 min. Finally, the temperature was maintained at 68 °C for the designated period (the pre-cultivation time). The growth of cells adhering to or just beneath the coverslip of the ATOC was observed with an Eclipse E600 optical microscope (Nikon, Tokyo, Japan) without phase contrast (Kuwabara and Igarashi 2012); the light intensity markedly decreased with phase contrast due to light interference resulting from the long light pathlength. The ATOC was placed on a Micro Heat Plate (MP-10DMH; Kitazato Supply Co. Ltd., Shizuoka, Japan), which had been modified by the manufacturer to cancel the electrical noise generated by periodic heating. When the Micro Heat Plate was set at 80 °C, the coverslip was at 65 ± 3 °C as determined using a surface thermometer, being cooled by ambient air in the room. A 40× objective and a 10× eyepiece were used for a total magnification of 400×, unless otherwise stated. Objectives requiring immersion oil, such as 100×, could not be used because of possible damage through heat transfer from the ATOC. Movies were recorded and processed as previously described (Kuwabara and Igarashi 2012) and cropped using the Ultimate video converter (Wondershare Software, Tokyo, Japan).

Phase-contrast and epifluorescence microscopy using the Live/Dead assay and the FM 1-43 dye (Life Technologies, Carlsbad, CA, USA) were performed using the same optical microscope, as described above. Images were captured and artificially colored as previously described (Kuwabara et al. 2011).

For TEM experiments, spheroid samples were fixed with 7.5% (w/v) glutaraldehyde in 0.2 M sodium cacodylate buffer (pH 7.2) for 2 h at 4 °C (Kuwabara et al. 2011). The concentrated fixative (7.5% glutaraldehyde) was used to preserve the spheroid OM as much as possible. We confirmed that the cell morphology was not altered in the fixative solution, and thus the distortion of spheroid OMs, as observed in some TEM results, may have occurred after fixation, plausibly during the process of dehydration. The fixed samples were processed as previously described (Kuwabara and Igarashi 2012) and examined using a JEM1010 (JEOL, Tokyo, Japan) or H-7650 (Hitachi, Tokyo, Japan) transmission electron microscope, operated at 80 kV.

The HTM movies and photographs, as well as the phase-contrast-microscopy, epifluorescence-microscopy, and TEM images presented are representative of results from at least 4 independent experiments in each case.

## Results

### Spheroid formation in the early growth phases

**Fig. 2:**
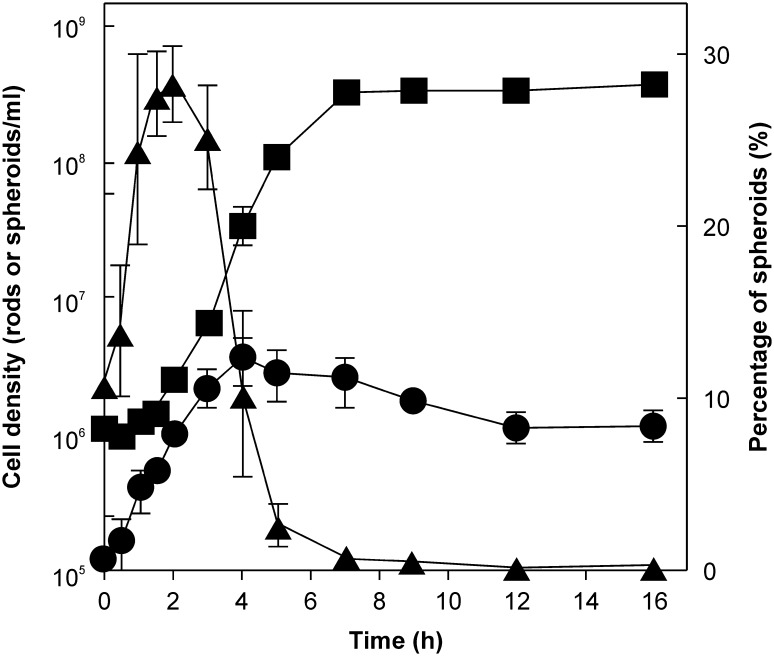
Growth curve of *Thermosipho globiformans*. Live rods and spheroids grown in batch cultures were directly counted by epifluorescence microscopy in Live/Dead assays. The* left* *y*-*axis* shows the densities of spheroids (*circles*) and rods (*squares*), while the* right* *y*-*axis* shows the ratio of spheroids to total cells (*triangles*)

When stationary-phase cultures were inoculated in fresh media, *T. globiformans* formed spheroids in the early growth phases (Kuwabara et al. 2011). The ratio of spheroids/total cells was maximal (~28%) at 2 h after starting batch cultivations, when inoculated at 10^6^ cells/mL (Fig. 2). Thereafter, the number of spheroids increased for up to 4 h post-inoculation, but that of rods increased more rapidly, resulting in a net decreased ratio of spheroids to total cells. Considering that spheroid formation is related to AL disintegration (Kuwabara et al. 2011), it is likely that some rods triggered AL disintegration in the stationary phase, while still retaining an overall rod-like morphology (e.g., Figure 2a in Kuwabara et al. 2011), and they transformed to spheroids after they started to grow in fresh media.

### Translocation of cells from rod moieties into OM balloons

Results from a previous TEM study indicated that partial inflation of the OM caused a knotty appearance of the rods, where the AL appeared to be loosened or partially disintegrated (Kuwabara et al. 2011). When rods with a knotty appearance were tracked during HTM after pre-cultivation for 1.5 h, the knotted structures abruptly inflated and formed balloons (Online Resource 1). The balloons were stained with FM1-43 (Kuwabara et al. 2011), suggesting that they contained the OM. These observations suggested that the OM was liberated from the AL, consistent with the previous finding that the spheroid OMs were not lined with thick ALs (Patel et al. 1985; Balk et al. 2002; Kuwabara et al. 2011), unlike rods (Kuwabara and Igarashi 2012).

Other HTM observations of 1.5 h-pre-cultivated cultures showed that knots on rods abruptly inflated to form balloons, followed by the immediate emergence of a few round cells (Online Resource 2). The abrupt formation of spheroids with multiple cells suggested that these cells formed in the rods (Kuwabara and Igarashi 2012) and later translocated into the balloons.

### Spheroid growth and formation of dish-shaped large cells

**Fig. 3:**
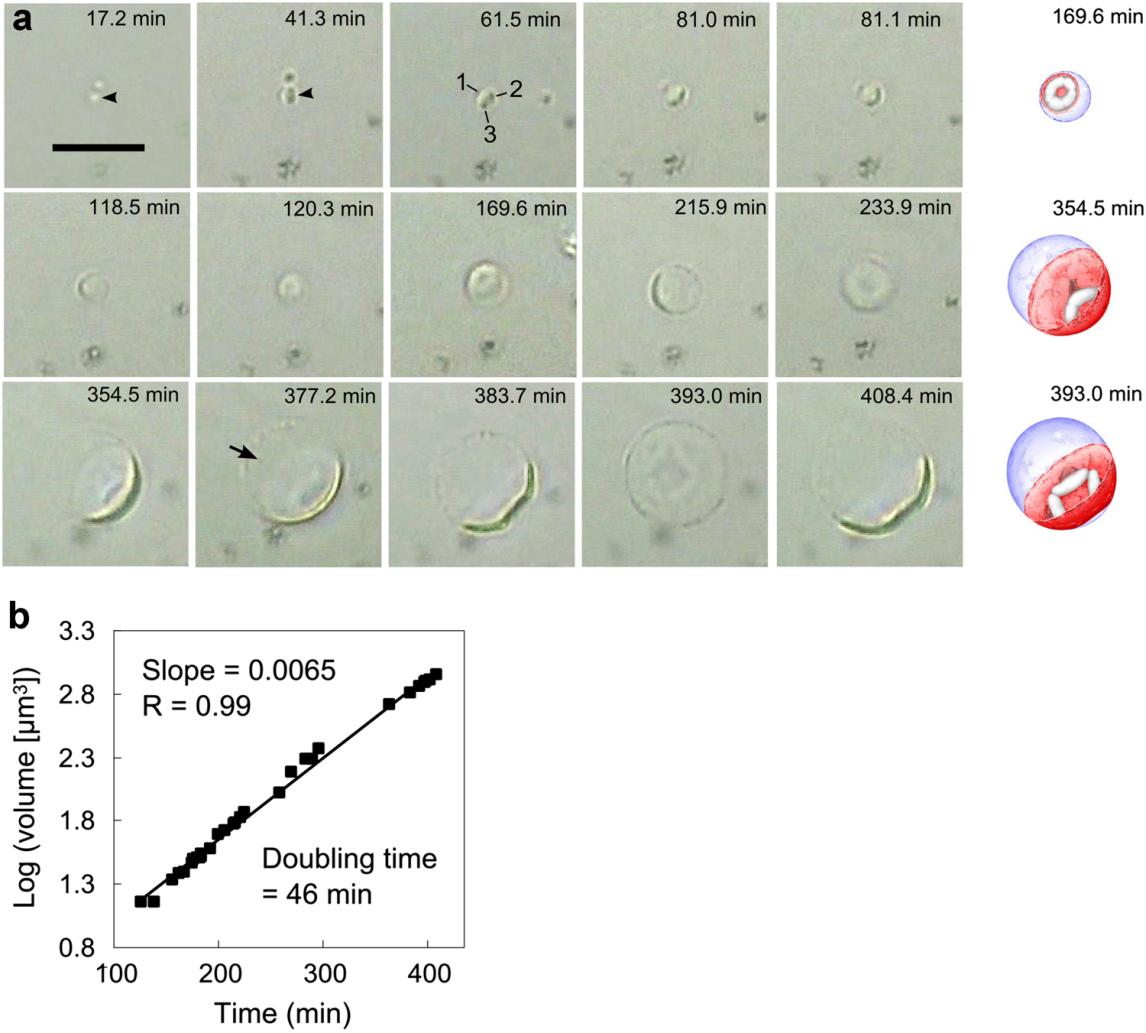
Growth of spheroids observed by HTM. An HTM movie was taken of a culture that was pre-cultivated for 1.5 h. The start of the observation period was defined as 0 min. **a** Snapshots at the indicated times are shown. An observed spheroid is indicated by an *arrowhead* (17.2 and 41.3 min), or is shown at the center of each panel. One elongated (1) and 2 globular cells (2, 3) are indicated in the image taken at 61.5 min. ‘Progenies’ rapidly moving in the periplasm were observed at 377.2 min (*arrow*). *Scale bar* 10 µm, applicable to all snapshots. Illustrations of specific spheroids are shown. Cells, nucleoids, and periplasms are shown in reddish, whitish, and purplish colors, respectively. Note that the illustrations at 169.6 and 354.5 min are copied from the respective photos, while that for 393.0 min shows an oblique image presumably taken from the side (383.7 and 408.4 min) and frontal (393.0 min) views. **b** Calculation of the volume-doubling time (=log 2/slope). Spheroid volumes were calculated from their diameters, assuming that the spheroid was spherical throughout. Snapshots in the early growth stages and those obtained under abnormal foci, both of which showed blurred outlines of spheroids, were omitted from the calculation. *R* correlation coefficient

To measure spheroid growth, we studied spheroids as small as possible, taking advantage of the bright appearance of cells at an upper focal plane in HTM (Fig. 3a, 17.2 min); in the normal focal plane, small spheroids were difficult to distinguish from non-living materials. The growth of spheroids, which were as small as 1–2 μm in diameter, was video-recorded in a fixed-vision field, until they migrated out of the field. The spheroid observed for the longest period is shown in Fig. 3. Initially, 1 elongated cell and at least 2 globular cells were observed in the spheroid (Fig. 3a, 61.5 min). All cells appeared elongated after an 81.0-min growth. Multiple cells may have appeared as a single elongated cell in a side-projection view (81.1 min). An enlarged cell was produced through an unidentified process (120.3 min, see Discussion), which showed an abnormal ring-like appearance (169.6 min). The spontaneous movement of spheroids showed a dish-shaped side view of the cell, whose bottom side was associated with the OM (215.9 min). An image taken at 233.9 min showed a frontal view of the cell located at the distal side of the spheroid. The spheroid having the dish-shaped cell further enlarged to as large as 12 μm in diameter (408.4 min). We observed ‘progenies’ moving in the periplasm (Kuwabara et al. 2011) at 377.2 min during the enlargement. Unfortunately, the ‘progenies’ shown in Fig. 3a were not obvious; thus, another movie showing the movement of ‘progenies’ is presented in Online Resource 3. Accompanying the enlargement of cells, the ring shape (169.6 min) transformed into a lip shape, which was observed in a frontal view of the cell at the distal side of the spheroid (393.0 min). The spontaneous movement helped us observe the 3D structure of the cell (Online Resource 4). At the end of Online Resource 4 movie, an image like that at 354.5 min (Fig. 3a) is shown, suggesting that the lip shape could have formed by that time. The bottom side of the dish-shaped cell with the lip-shaped structure appeared to be detached from the OM (393.0 min between 383.7 and 408.4 min).

In different HTM observations, ‘progenies’ moving in a spheroid periplasm, similar to those observed at 377.2 min (Fig. 3a), were successfully video-recorded (Online Resource 3); focusing was difficult because of their small size and movements toward the depth direction. Similar miniscule substances were identified as ‘progenies’ in a previous study; they were stainable with the Live/Dead assay and with FM 1-43, indicating that they contained nucleic acids and a membrane (Kuwabara et al. 2011).

The volumes of spheroids were estimated by measuring their diameters, assuming that they were spherical throughout. The volume-doubling time was estimated as 46 min for spheroid diameters ranging from 3.0 to 11.9 μm, which were measured in 30 images (Fig. 3b). Similar measurements for the 5 other spheroids gave similar results (Table 1). It should be noted that no spheroids fissioned during the growth period.

**Table 1:** Growth of *Thermosipho globiformans* spheroids studied by HTM

Observation	Spheroid	Diameter range (µm)	Doubling time (min)	Correlation coefficient	Data points^a^
1	1	3.0–11.9	46	0.99	30
	2	2.8–6.4	46	0.99	12
	3	3.2–5.1	49	0.98	7
	4	3.8–4.8	42	0.96	6
2	5	10.0–15.9	47	0.98	18
	6	8.2–12.9	44	0.97	14

The results of 2 HTM observations are summarized. The data for spheroid 1 are from Fig. 3b

^a^Number of data points used to calculate doubling times

### Phase-contrast-microscopy and epifluorescence-microscopy observations

**Fig. 4:**
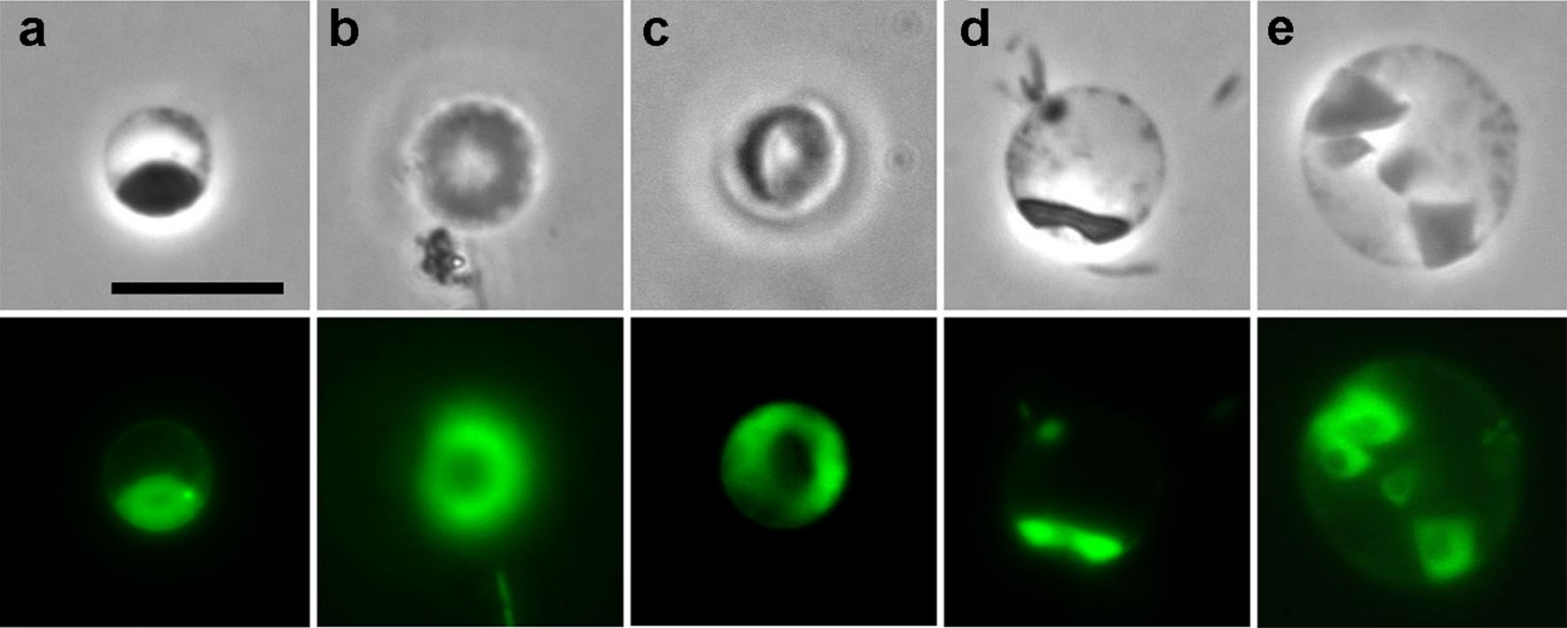
Various nucleoid morphologies of dish-shaped cells. Morphologies observed in the exponential growth phases are shown according to the progression of spheroid growth. Phase-contrast (*upper panels*) and epifluorescence (*lower panels*) micrographs observed in Live/Dead assays are shown. Spheroids with a dish-shaped cell having a possible precursor of a ring-shaped nucleoid (**a**), a ring-shaped nucleoid (**b**), a lip-shaped nucleoid (**c**), a possible lip-shaped nucleoid (**d**), and a spheroid with multiple cells having ring-shaped nucleoids (**e**). Note that the spheroid OMs were unfocused when the nucleoids were focused in the frontal views (**b, c**) and that a rod was photographed together with a spheroid (**b**, *lower center*). The ‘satellite cells’, defined as medium-sized globular cells bound to the inner surface of spheroid OM (**d**), might represent large ‘progenies’ arrested on the OM and/or cells that failed to grow, as the dish-shaped cells did. *Scale bar* 10 µm (*all panels*)

A lip shape, similar to that observed by HTM (Fig. 3a, 393.0 min), has been previously observed by epifluorescence microscopy in Live/Dead assays (Kuwabara et al. 2011), suggesting that the morphology represented a nucleoid because the Live/Dead assay is used to stain nucleic acids. If this possibility is correct, then the ring shape shown in Fig. 3a (169.6 min) was also likely of nucleoid origin, since it transformed to the lip shape (Fig. 3a, 393.0 min). We found ring-shaped and lip-shaped nucleoids in similar growth stages in batch cultures (Fig. 4b, c). Furthermore, careful examination of the epifluorescence images revealed nucleoids whose central portions were slightly dark when compared to the outer portions (Fig. 4a). This type of nucleoid is likely to be a precursor of ring-shaped nucleoids. The epifluorescence-microscopy observations and the HTM results (Fig. 3a) collectively suggested that the nucleoids reorganized during growth, as represented by the morphological changes. It should be noted that the intensity of Live/Dead staining of spheroid nucleoids was far stronger than that of rods (for example, see Fig. 4b), suggesting that the spheroids were growing actively, consistent with the spheroid growth shown in Fig. 3. When the spheroid of Fig. 4c is viewed from the lower left side, the image would be similar to the image shown in Fig. 4d, consistent with the lip-shaped nucleoids occurring in the cells whose bottom sides were detached from the OMs (Fig. 3a, 393.0 min); the central portions of the nucleoids were faintly stained compared to the staining observed along the edges (Fig. 4d). When plural dish-shaped cells were present in a spheroid, plural ring shapes were observed (Fig. 4e). However, we never observed plural lip shapes in a spheroid.

Because ‘progenies’ could be observed at 400× magnification by HTM, they likely grew to a relatively large size; in a previous study (Kuwabara et al. 2011), 1000× magnification was necessary to observe ‘progenies’ of approximately 0.5 μm in length. We also observed large ‘progenies’ (approximately 1 μm in length) in batch cultures in growth stages similar to those used for the HTM-based observations (Fig. 3a, 377.2 min), which were moving in the periplasm (Online Resource 5). The ‘progenies’ tended to stop moving after binding to the spheroid OM. Without such movement, they could not be distinguished from cells that had translocated from rod moieties but had not enlarged, as observed with the cell shown in the upper left region of the spheroid periplasm shown in Online Resource 5.

HTM failed to show spheroids with in-periplasm rod formation; this failure could have been due to the sedimentation of grown spheroids from just beneath the coverslip of the ATOCs. Therefore, we batch-cultured *T. globiformans* for 16 h, sampled the cultures from the bottoms of the serum bottles without agitation, and analyzed them using the Live/Dead assay. We observed spheroids with in-periplasm, moving, rod-like cells, whose fluorescence intensities matched those of mature rods outside the spheroids (Online resource 6).

### TEM observations

**Fig. 5:**
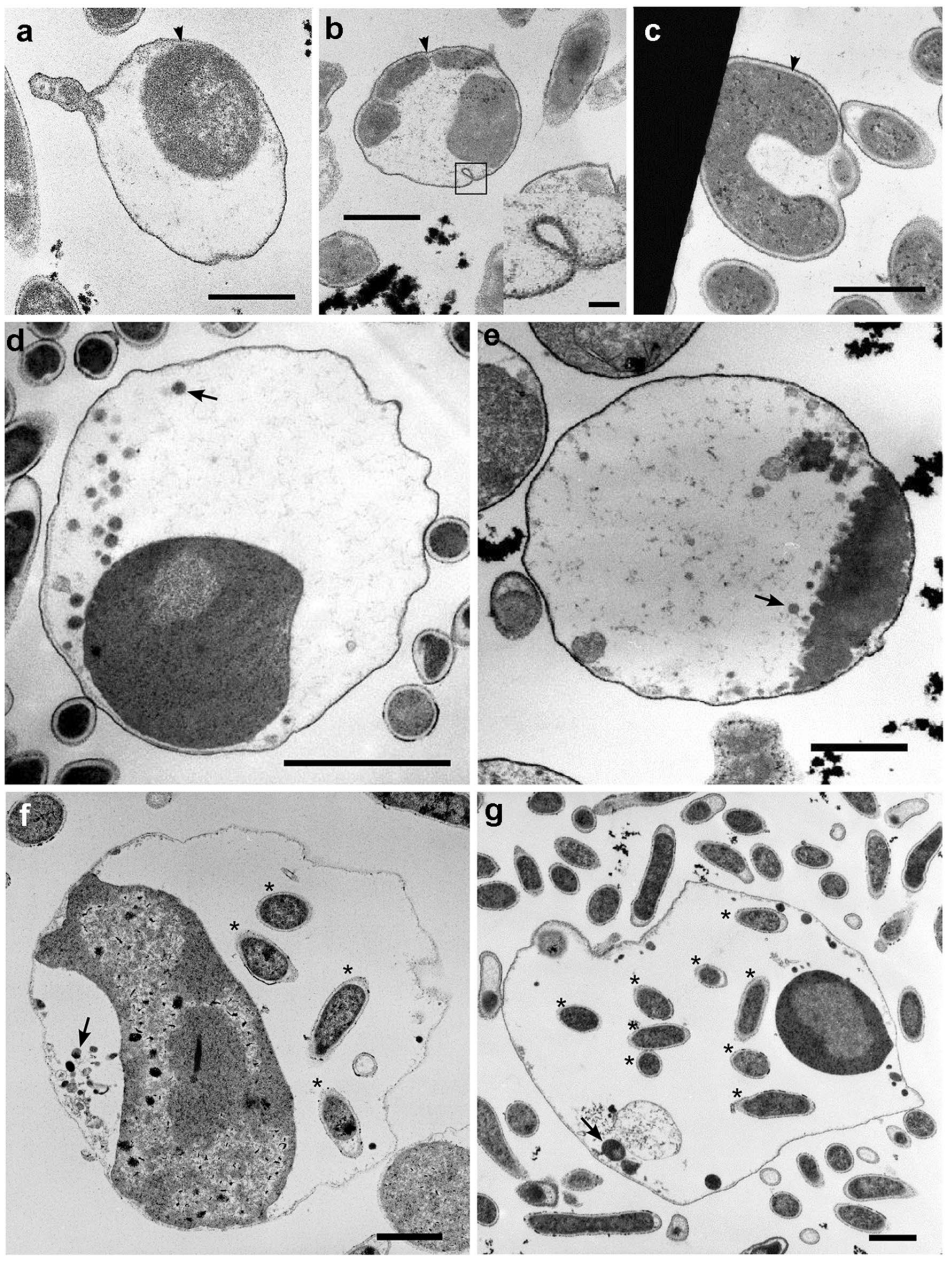
Fine structures of spheroids formed during the process of progeny production. A spheroid into which a rod-shaped and subsequent small cells could translocate (**a**). A spheroid with multiple cells, which elongated to various degrees, with an inset emphasizing the invagination of a spheroid OM (**b**). A spheroid showing a side view of a dish-shaped cell (**c**). Spheroids showing a dish-shaped cell, vesicles, and/or rods in the periplasm (**d–g**). Note that an AL-like thin layer between the OM and the CM (a*rrowheads*) is obvious in panels **a–c.** The *asterisks* indicate progeny rods, while *arrows* indicate relatively large vesicles in each spheroid (**d–g**). Note that the *scale bars of panels* **e–g** are shorter than that in *panel* **d**. *Scale bars* (**a–g**), 1 µm; *inset* in (**b**), 100 nm

Next, we studied the fine structures of spheroids during the course of their growth. The translocation of cells from rods into spheroids (Fig. 5a) and the elongation of multiple cells in the spheroids (Fig. 5b) were also suggested by our TEM results. Side views of dish-shaped cells did not show septation traversing the cells (Fig. 5c), consistent with fusions of multiple cells, although this cannot exclude the possibility of dish-shaped cell formation from single cells. As observed previously (Kuwabara et al. 2011), the spheroids lacked a thick AL (Fig. 5). The observed invaginations of spheroid OMs (Fig. 5b) suggested the absence of a TEM-invisible rigid structure that lined the OM. However, a thin layer, possibly a remnant AL, was present between the closely associated OM and CMs (Fig. 5a–c, arrowheads).

When spheroids showing ‘progenies’ were analyzed by TEM, several rounded/ellipsoidal vesicles were observed in the periplasm (Fig. 5d–g; arrows). The rough surfaces of the dish-shaped cells showed vesicle-like structures (Fig. 5e), suggesting that they produced the vesicles. In some cases, rods (namely, with togas) were observed in the periplasm (Fig. 5f, g), consistent with the in-periplasm, rod-like cells observed with an epifluorescence microscope (Online resource 6). The large rounded/ellipsoidal vesicles resembled the cells of the rods (Fig. 5g), which would be situated in different angles with respect to the plane. These results suggested that the TEM-observed vesicles represented immature progenies, which were identical to the optical microscopy-observed ‘progenies’ that grew to acquire togas and became rods. The overall cell cycle is summarized in Fig. 1.

### Rupture and regeneration of OM

**Fig. 6:**
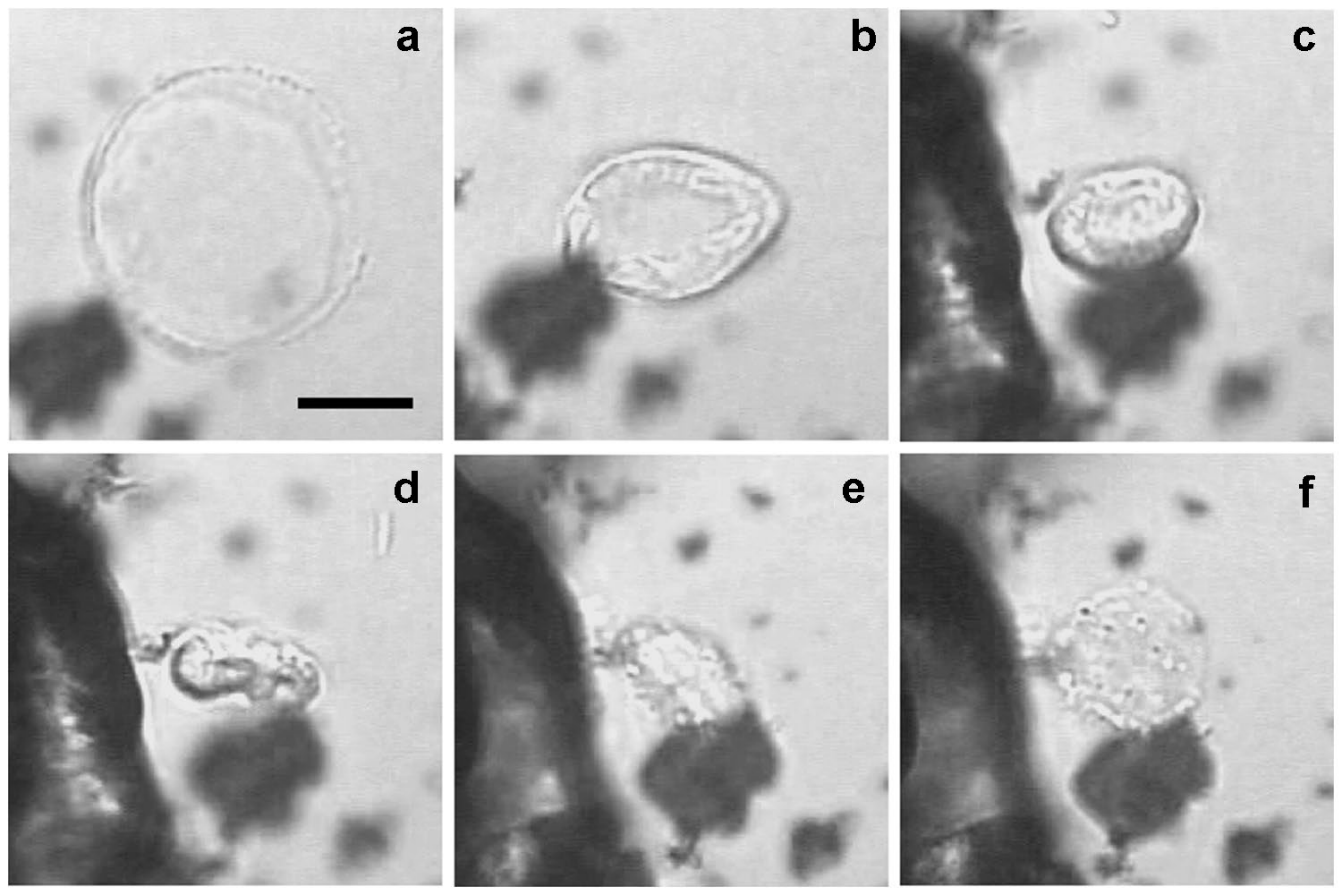
Rupture and regeneration of the spheroid OM. An HTM movie was made for a culture pre-cultivated for 9.5 h. The presented scenes reflect observations from 2.5 h after starting HTM. Snapshots of the movie, just before the rupture of the spheroid (**a**), which show a frontal view of a dish-shaped cell expanded at the distal side of the spheroid, and at 0.8 s (**b**), 17 s (**c**), 2.5 min (**d**), 2.8 min (**e**), and 17 min (**f**) post-rupture. *Scale bar* 10 µm (all panels)

The OMs of spheroids with dish-shaped cells eventually ruptured (Fig. 6). During the subsequent shrinkage of spheroid OMs, the cells showed a lip-like appearance by HTM (Fig. 6b), indicative of the presence of rigid lip-shaped structures in the cells. The rupturing appeared to facilitate the release of ‘progenies’ from the periplasm. However, they were not detected in precipitates following differential centrifugation of stationary phase cultures. This finding suggested that the ‘progenies’ bound to mature cells and cosedimented, or died following their release. Surprisingly, after the rupture, OM-like structures slowly inflated again (Fig. 6), suggesting that the cells were stable and OM formation continued even after its rupture. This finding was consistent with the previous observation that toga formation is uncoupled from cell growth at late phases (Ranjit and Noll 2016).

## Discussion

The data shown in Fig. 2 suggested that *T. globiformans* was comprised of at least 2 subpopulations of rods in the stationary phase, one of which triggered AL disintegration but retained their overall rod-like morphologies. The present findings showed that the subpopulation transformed to spheroids and contributed to in-periplasm progeny production in fresh media. The remaining rod subpopulations, which did not trigger AL disintegration, produced AL in fresh media and could proliferate by binary fission (Fig. 2). It is presently unknown to what degree progeny production in spheroid periplasms contributes to the total bacterial population.

What determines whether *T. globiformans* proliferate via binary fission or in-periplasm progeny production is currently unknown. Spheroid formation was initiated by inflation of OM, which was previously associated with AL (Kuwabara et al. 2011). The AL and PGs share similarities in terms of their intracellular localization and functions in maintaining cell morphology (Lam et al. 2009; Leaver et al. 2009; Kuwabara et al. 2011). Because inactivation of the *murE* operon (Leaver et al. 2009), which contains genes required for murein synthesis, and *mrcA* (Lam et al. 2009), encoding penicillin-binding protein 1A, cause spheroid formation, it is plausible that AL disintegration also leads to spheroid formation. Because spheroid formation is a prerequisite for in-periplasm progeny production, AL disintegration could be a remote cause of progeny production by spheroids. In contrast, rods undergoing binary fission necessarily have togas containing thick ALs (Kuwabara and Igarashi 2012), and thus, the presence and absence of AL-forming activity are likely causes of binary fission and in-periplasm progeny production, respectively.

A limitation of this study is that we did not clarify how dish-shaped cells formed in spheroid periplasms. The most likely mechanism is that the dish-shaped cells formed through the fusion of elongated cells (Fig. 5b, c); the elongated cells observed beforehand could not be detected after the dish-shaped cells formed (Fig. 3). The possible in-periplasm fusion event appears similar to the dynamin-dependent fusion of mitochondria (van der Bliek et al. 2013). Interestingly, *Thermosipho africanus* (Nesbø et al. 2009), which is phylogenetically the nearest species to *T. globiformans* (Kuwabara et al. 2011), possesses a homolog (NCBI: WP_004102778.1) of a GTPase domain of the dynamin-like protein (NCBI: CUB38200.1) of *Bacillus subtilis* (Bürmann et al. 2011). This dynamin-like GTPase could facilitate the fusion of bacterial cells. The fusion event could not be clearly demonstrated in the present study because of the low resolution of HTM. The low magnification and resolution of HTM also hampered a direct observation of the ejection of immature progenies from spheroid periplasms. The in-periplasm formation of dish-shaped cells and the ejection of immature progenies should be studied further using a computer-assisted optical microscope, which provides higher resolution (Hatano et al. 2007).

The immature progenies produced in spheroid periplasms appeared too small to have a complete genome; all Thermotogales species with fully sequenced genomes have a genome size of approximately 2 MB (http://www.kegg.jp/kegg/catalog/org_list.html). If each immature progeny has only a genome fragment, but not a complete genome, complementation must occur to make the genome complete. Thermotogales bacteria are known to be highly capable of horizontal gene transfer (Nelson et al. 1999; Nesbø et al. 2009; Zhaxybayeva et al. 2009), which could potentially serve to complement possible genetic deficiencies in immature progenies. In such a scenario, only progenies that by chance had the complete genome would grow to form rods, consistent with results showing that the number of ‘progenies’ markedly decreased along with the emergence of the rods [compare Movies S1, S2 in Kuwabara et al. (2011) with Online Resource 6]. Another possibility is that the fusion of ‘progenies’ could make the genome complete.

The AL disintegration-derived cell enlargement and intracellular vesicle (immature progeny) formation could provide hints as to how cells became enlarged and formed an endomembrane during eukaryogenesis; recently, symbiont α-proteobacterial outer membrane vesicle-derived endomembrane formation was proposed (Gould et al. 2016). Although the genomes of Thermotogales are unrelated to those of eukaryotes, understanding the mechanisms of these cell-biological characteristics may help reveal mechanisms of cell enlargement and endomembrane formation during eukaryogenesis. It is interesting to study the effects of horizontal transfer of archaeal genes to the *T. globiformans* genome.

In the present study, the growth dynamics of *T. globiformans* were partially revealed by the combination of HTM, phase-contrast microscopy, epifluorescence microscopy, and TEM. Single-cell growth studied using HTM showed that spheroids became enlarged by more than 300-fold without fission and produced ‘progenies’, and the other modes of microscopy in combination showed that spheroids produced ‘progenies’ or immature progenies, as well as rods in the periplasm. These findings represent a novel aspect of Thermotogales proliferation. Although *T. globiformans* is the only known example of Thermotogales bacteria that exhibit in-periplasm progeny production at present, we recently observed production of ‘progenies’ by *Thermotoga maritima* (Huber et al. 1986). To what extent this strategy is used by Thermotogales and the consequences of this strategy should be clarified in future studies.

## Electronic supplementary material

Below is the link to the electronic supplementary material.

Online Resource 1. Abrupt formation of a balloon on a rod, which is shown with an arrow. An HTM movie was made of a culture that was pre-cultivated for 1.5 h, using a 60× objective. The presented scene was observed at 10 min after starting HTM. Scale bar = 10 µm (MPG 2595 kb)

Online Resource 2. Translocation of cells from rod moieties into a balloon, which is shown with an arrow. An HTM movie was made of a culture that was pre-cultivated for 1.5 h. The presented scene was observed at 70 min after starting HTM. Scale bar = 10 µm (MPG 2576 kb)

Online Resource 3. HTM observation of ‘progenies’ moving in a spheroid periplasm. ‘Progenies’ are indicated by an arrow at the initial scene. Viewing the movie at full screen may help readers observe the movement. An HTM movie was made of a culture that was pre-cultivated for 3 h. The presented scene was observed at 6 h after starting HTM. Scale bar = 10 µm (MPG 2099 kb)

Online Resource 4. Movement of a spheroid having a dish-shaped cell with a lip-shaped nucleoid. The movie source is the same as that shown in Fig. 3. The speed of the movie is 20× that of the original. Scale bar = 10 µm (MPG 5803 kb)

Online Resource 5. Large ‘progenies’ in a spheroid periplasm. A movie was made of a culture that was batch-cultivated for 6 h and stained in a Live/Dead assay. Scale bar = 10 µm (MPG 1524 kb)

Online Resource 6. Rod-like progenies in a spheroid periplasm. A movie was made of a culture that was batch-cultivated for 16 h. The sample was taken from a bottom part in the serum bottle without homogenization and stained in a Live/Dead assay. Scale bar = 10 µm (MPG 4194 kb)

## Abbreviations

AL: Amorphous layer
ATOC: Anaerobic thermophile observation chamber
CM: Cytoplasmic membrane
HTM: High-temperature microscopy
OM: Outer membrane
PG: Peptidoglycan
TEM: Transmission electron microscopy
